# YTHDF1 Regulates RELA m6A Modification and Activates the NF‐Kappa B Signaling Pathway to Promote the Mechanism of Gastric Cancer

**DOI:** 10.1002/cam4.70611

**Published:** 2025-01-17

**Authors:** Yangyuan Huang, Shihao Liang, Liping Li, Qingyu Zeng, Bin Li

**Affiliations:** ^1^ Department of Gastroenterology Affiliated Hospital of Guilin Medical University Guilin Guangxi People's Republic of China; ^2^ Guangxi Key Laboratory of Molecular Medicine in Liver Injury and Repair Affliated Hospital of Guilin Medical University Guilin Guangxi People's Republic of China

**Keywords:** gastric cancer, NF‐kappa B signaling pathway, RELA, YTHDF1

## Abstract

**Background:**

Gastric cancer (GC) is an important cause of death. Molecular targeted therapy and immunotherapy are progressing rapidly. It is very important to explore the pathogenesis pathways of GC and provide strong support for its treatment. However, the mechanism of occurrence and development of GC is still unclear.

**Methods:**

Online databases and immunohistochemistry (IHC) of clinical samples were used to analyze the differential expression of YTHDF1 in the GC and nearby tissues, and its effect on survival prognosis. In vitro experimental study of GC, other mechanisms and functional analyses were specifically designed and performed too.

**Results:**

Online data and clinical samples analysis showed that the expression of YTHDF1 in GC was markedly elevated compared to surrounding tissues. Higher YTHDF1 levels were correlated with worse survival outcomes. Analysis of correlation with clinical parameters showed that the expression level of YTHDF1 exhibited a favorable correlation with lymphatic metastases, as well as with PD‐1 and PD‐L1 levels. In vitro studies of YTHDF1 overexpression have demonstrated its ability to enhance GC cell growth and migration while inhibiting apoptosis. Based on our results, RELA is a downstream target of YTHDF1, and YTHDF1 triggers the NF‐κB signaling pathway by regulating RELA translation.

**Conclusion:**

In comparison to adjacent tissues, GC exhibits significantly elevated YTHDF1 expression. Increased YTHDF1 expression in the GC is correlated with decreased patient survival. Lymph node metastasis and the expression of PD‐1 and PD‐L1 are positively correlated with YTHDF1 levels. YTHDF1 inhibits apoptosis while promoting the migration and proliferation of GC. Additionally, it stimulates the NF‐κB pathway and controls the translation of RELA.

## 1 Background

Gastric cancer (GC) is a common malignant tumor and an important cause of death. Gastrointestinal cancer represents 25% of all reported cancer cases worldwide and is responsible for 33% of cancer‐related fatalities [1]. Based on the GLOBOCAN 2020 database of the International Agency for Research on Cancer (IARC), GC represents 5.6% of all tumors and has a mortality rate of 7.7% [2]. There has been rapid progress in the treatment of GC through molecular targeted therapy and immunotherapy. Thus, it is very important to explore the pathogenesis pathways of GC and provide strong support for its treatment which is expected to achieve a major breakthrough.

An assortment of RNA chemical modifications found in organisms have been identified [3], and they have a significant impact on the onset and progression of illnesses, particularly cancers. Out of all the chemical modifications, the most prevalent, plentiful, and conservative internal alteration is N6‐methyladenosine (m6A) in eukaryotic messenger RNA (mRNA), microRNA (miRNA) [4], and long noncoding RNA (lncRNA) [5], which involves adding a methyl group to the sixth position of adenosine. m6A consists of three distinct components and is synthesized by m6A methyltransferases (writers), purged by erasers, and recognized by m6A‐binding proteins (readers) [6]. “Writers” that stimulate methylated include METTL14, METTL5, METTL3, ZC3H13, WTAP, VIRMA and RBM15. “Erasers” are demethylases, which include ALKBH5 and FTO. “Readers” are methylated reading proteins unique to m6A; they include ELAVL1, YTHDF1/2/3, and IGF2BP1/2/3. Dysregulation of these three kinds of regulatory proteins is frequently observed in cancer. By exerting control over various downstream molecules and signaling pathways, they influence the progression of cancer and the prognosis of patients by either promoting or inhibiting the disease [7]. “Readers” are a class of proteins that contain YTH functional domains, which are highly conserved and exist in 174 different proteins in eukaryotes. They consist of 145 amino acids, which are arranged in sequences located between the unstructured N‐terminal and C‐terminal regions [8]. Proteins with functional domains participate in a variety of biological functions by affecting the expression of downstream genes, which include RNA splicing, stability, nuclear transport, regulation, degradation, and translation [9, 10, 11, 12]. The YTH domain's secondary structure comprises six β‐chains and four α‐helices. These chains exhibit selectivity towards YTH domain proteins that encapsulate single‐stranded unstructured RNA in order to identify m6A‐binding sites via a conserved aromatic family that includes three tryptophan residues [13]. YTHDF1 binds to mRNA with m6A modification and induces its translation. YTHDF2 enhances the degradation of its mRNA target by enlisting the CCR4‐NOT deadenylating complex, whereas YTHDF3 stimulates translation and accelerates the degradation of its mRNA target [14].

The physiological functions of YTHDF1 proteins vary across different tumors, which suggests their specificity to certain cellular environments. Numerous reports have highlighted the differential expression and functional roles of YTHDF1 in tumor development. For example, in breast cancer research, YTHDF1 recognizes and binds to FOXM1 mRNA through m6A modification, thereby facilitating the translation process of FOXM1 and promoting metastasis [15]. Prostate cancer research has provided evidence that YTHDF1 directly targets Polo‐like Kinase 1 (PLK1). By altering m6A, YTHDF1 identifies PLK1 mRNA and increases its efficacy of translation; this facilitates the pathway overactivation of phosphatidylinositol 3 kinase/protein kinase B (PI3K/AKT) [16]. Furthermore, there is an intense connection between the expression of YTHDF1 and the malignant characteristics of head and neck squamous cell carcinoma (HNSCC) tumors. Significantly, this connection exhibits a positive relationship with CD4^+^ T cells while displaying a negative relationship with CD8^+^ T‐cell infiltration, thereby indicating an association between YTHDF1 and the tumor microenvironment (TME) [17, 18]. This suggests that YTHDF may serve as a predictive marker for guiding immunotherapy. Researchers have evaluated the clinical relevance of m6A modification in GC patient cohorts while identifying pathways and phenotypes regulated by this modification. These findings suggest a pro‐cancer role for YTHDF in GC; however, the underlying mechanism remains unknown. Therefore, further investigation into the role of YTHDF in GC is warranted.

The expression regulator of κ‐light‐chain genes in B lymphocytes, NF‐κB, has five members in its family: NF‐κB1/p50, NF‐κB2/p52, c‐REl, RELB, and RELA/p65 [19, 20]. These highly homologous proteins interact with the inhibitor IkBα to modulate functional gene expression and thereby influence cellular behavior [21]. RELA plays a critical role as a transcriptional component in the NF‐κB pathway, promoting the upregulation of proinflammatory and carcinogenic molecules that contribute to tumor development and treatment resistance. Ultimately, this activation leads to cell proliferation, apoptosis, tumor metastasis, proinflammatory response, and metabolic reprogramming, which drive tumor progression [22, 23].

Based on the previously provided background, the bioinformatics investigation showed a significant difference in YTHDF1 transcription between malignancy and tissue normalcy. Furthermore, a positive correlation existed between YTHDF1 and RELA in GC. Subsequently, our objective was to validate the association of YTHDF1 with both neoplastic and adjacent tissues through pathological tissue microarray detection and to analyze its correlation with clinical parameters. Simultaneously, our objective was to examine the impact of YTHDF1 on GC cells using in vitro tests and to uncover the underlying molecular route.

## 2 Methods

### 2.1 Tissue Samples and Immunohistochemistry (IHC) Staining

The human pathological tissue chip from GC was purchased from Shanghai Xinchao Co Ltd. (No.: HStmA180Su19), with 180 original spots. The tissue chip included a total of 55 couples of tumors and their corresponding surrounding tissues, as well as 13 individual cancer tissues. After receiving signed written informed permission, the patients' private information was erased (Table S1: Gastric Cancer Pathology Tissue Microarray HStmA180Su19 Data). YTHDF1 expression was detected by IHC analysis. After sample dewaxing, rehydration, antigen retrieval, and blocking, the chip was incubated with antibodies against YTHDF1 at 4°C overnight. Hematoxylin was used as a counterstain after DAB development was applied to the slides. The slides with color were examined through a microscope, and photos were captured using the DefaultGroup software controller.

### 2.2 GC Cell Lines, Plasmids, and Antibodies

Guangxi Key Laboratory of Molecular Medicine in Liver Injury and Repair provided the cell lines MGC‐803 and HGC‐27 in 2021. These cell lines were confirmed to be devoid of mycoplasma contamination and had a maximum utilization period of 6 months. All cells were propagated up to a maximum of 25 passages. We utilized DMEM (Gibco, NY, USA) for MGC‐803 and 1640 (Gibco) media for HGC‐27. The cell lines were maintained in an arrangement consisting of 10% fetal bovine serum (Gibco) and 1% penicillin–streptomycin (Solarbio, China) at 37°C in a cell‐culture incubator with 5% CO2. Stably transfected cell lines were obtained by gene transfection using PolyJet transfection reagent in this experiment and screening using puromycin (Solarbio, China). Plasmid pGFP‐C‐OEYTHDF1 was purchased from Shanghai Genechem Co. Ltd.; pRFP‐C‐shRELA was purchased from Chongqing Unibio Co. Ltd.; and Tiangen Biotech Co. Ltd. (Beijing, China) supplied the *Escherichia coli* ( *E. coli* ). YTHDF1 (rabbit, Abcam, UK), YTHDF1 (rabbit, ABclonal, MA, USA), RELA (rabbit, ABclonal), and GAPDH (rabbit, Abcam) were the commercial antibodies used.

### 2.3 Cell Plasmid Transfection

We waited until the cell density reached 70%–80% confluence in a six‐well plate. The entire medium was replenished with 2 mL of fresh complete culture medium 30–60 min prior to transfection. For each well of the six‐well plate, we diluted 2 μg of DNA into 100 μL of serum‐free medium and mixed it by gently pipetting. We added 3 μL of PolyJet transfection reagent to 100 μL of serum‐free medium per well and mixed gently by pipetting 3–4 times. We added the serum‐free medium containing PolyJet transfection reagent to the serum‐free medium mixed with plasmid, and mixed it by gently pipetting 3–4 times. The solution was added dropwise and evenly to the six‐well plate after incubation for 15–30 min. After 5 h of transfection, the medium was replaced. At 24–48 h after the cells were transfected, the transfection fluorescence was observed under a microscope; cellular RNA was extracted at 48 h, and cellular protein was extracted at 72 h for verification.

### 2.4 RNA Extraction and qRT‐PCR Analysis

The process of extracting total RNA from cells and tissues included the use of TRIzol (Invitrogen) and a phenol/chloroform‐based technique, following the instructions provided by the manufacturer. Extract a cell sample from a 6‐well plate, add 1 mL TRIzol, mix well and let stand for 5–10 min. Add 0.2 mL chloroform, mix vigorously, let stand for 2–3 min, then centrifuge at 4°C for 10 min. Transfer the upper aqueous phase to a new tube, add an equal volume of isopropanol, let stand for 10 min, then centrifuge for 10 min. Collect the RNA pellet. Wash with 75% ethanol, centrifuge to remove ethanol, allow to air dry, finally resuspend in RNase‐free water and store at −80°C. Monad reverse transcription reagent was used for the reverse transcription process. The target sequence information in the NCBI database was used to design primers using a primer design tool (NCBI, USA). The powerUP SYBR Green Master Mix (Applied Biosystems, USA) was used for qRT‐PCR using the CFX96 Touch real‐time fluorescence quantitative PCR detection system (Bio‐Rad), and the final reaction volume was 20 μL. Pre‐denaturation at 50°C for 2 min, denaturation at 95°C for 2 min, annealing at 60°C, and extension for 1 min, a total of 40 cycles. The primers used were (5′‐3′) YTHDF1 forward primer AGGCTTTCACAGCGACAC, YTHDF1 reverse primer CTGGCATGTTCACATTTGTC, RELA forward primer GGACTACGACCTGAATGC, RELA reverse primer CAAAGATGGGATGAGAAAG GAPDH forward primer TGCACCACCACTGCTTAGC, GAPDH reverse primer GGCATGGACTGTGGTCATGAG. All reactions for each sample were performed in triplicate, with GAPDH serving as the reference gene for normalization. Employ the 2^−ΔΔCt^ technique. A *t*‐test was used to compare gene expression levels.

### 2.5 Western Blot Analysis

A protein sample is extracted from a 6‐well plate, and its concentration is determined. The protein is separated by SDS‐PAGE electrophoresis and transferred to a PVDF or nitrocellulose membrane, followed by blocking of non‐specific binding sites. After adding and incubating with the primary antibody, the membrane is washed. Then, the secondary antibody is added and incubated, followed by another wash. Finally, protein signals are detected using chemiluminescence or color development, and the results are recorded.

### 2.6 Colony Formation Assay

Inoculate 500–1000 cells per well in a 6‐well plate. After the cells have formed a colony, gently remove the medium and wash the cells with phosphate‐buffered saline (PBS) to remove any remaining medium. Then, add 0.5% crystal violet dye and let it stand for 10–30 min to stain the cells. After staining, rinse again with PBS to remove excess dye. Finally, observe and record cell colonies with more than 50 cells in each well to evaluate the proliferative capacity of the cells.

### 2.7 Cell Viability Assay (CCK‐8 Assay)

After digesting the cells, they need to be resuspended and counted using a cell counter. Afterwards, a total of 5000 cells were distributed into each well of a 96‐well plate, while 4 additional wells were prepared as duplicates. At 24, 48, 72, and 96 h, add 10 μL of CCK‐8 solution and place it in the cell culture incubator for a further 2 h. Subsequently, the 96‐well plate was taken out and the absorbance value was measured at 450 nm using a microplate reader.

### 2.8 Transwell Migration Experiment

In the upper chamber of the migration chamber, 200 μL of fetal bovine serum (FBS)‐free medium containing 50,000 cells was dropped. We added 600 μL of 20% FBS medium to the lower chamber of the 24‐well plate. We then placed the 24‐well plate in a cell incubator and continued culturing for 24 h. The supernatant was removed, and the chamber was delicately rinsed three times with PBS. Then, 400 μL of paraformaldehyde was added to each well of the 24‐well plate to fix the cells for 1 h, and the fixation solution was removed by a suction method. The chamber was soaked in the crystal violet solution, and then the crystal violet in the upper chamber was washed away with clean water overnight. A cotton swab was used to wipe the bottom of the chamber, and we finally observed the migration of GC cells under an inverted microscope and took photographs for storage.

### 2.9 Migration Assay

A linear abrasion (“wound”) was produced in the monolayer with a sterile p10 pipette tip, 48 h after transfection. Cellular debris was eliminated by cleaning with PBS, followed by the addition of serum‐free medium. At 0 h, an inverted microscope was used to photograph the distance, and 48 h later, the same position was photographed again to compare and observe cell migration. To enhance the clarity of the figures, the bright‐field photos were flipped using ImageJ.

### 2.10 Flow Cytometry

The cells were cultivated in a 12‐well plate. Following two washes with PBS, the cells were enzymatically dissociated using EDTA‐free trypsin. Upon achieving a rounded morphology and partial shrinkage, the trypsin digestion was terminated by adding medium containing 10% serum. Subsequently, the cells were dislodged using a pipette and collected through centrifugation at 300 × *g* for 5 min at 4°C. The cells were washed twice with precooled PBS and centrifuged at 300 *g* for 5 min at 4°C each time. We removed the PBS by aspiration and then added 100 μL of 1x binding buffer to reconstitute the cell suspension. Next, we introduced 5 μL of Annexin V‐AlexaFluor647 and 10 μL of PI into the solution. We then gently combined the mixture and stored it in a light‐protected environment at room temperature for 15 min. We included 400 μL of 1x binding buffer, thoroughly mixed it, and then placed the resulting mixture on ice. The samples were detected with a flow cytometer within 1 h.

### 2.11 MeRIP‐Seq and RNA Immunoprecipitation (RIP)

MeRIP‐seq was performed by Cloudseq Biotech Inc. (Shanghai, China). Briefly, m6A RNA immunoprecipitation was performed with the GenSeqTM m6A RNA IP Kit (GenSeq Inc., China) in line with the manufacturer's instructions. The library quality was evaluated with BioAnalyzer 2100 system (Agilent Technologies Inc., USA). Library sequencing was performed on Illumina Hiseq instrument with 150 bp paired‐end reads. RIP assays were conducted using a Magna RIP RNA‐Binding Protein Immunoprecipitation Kit (Millipore, USA) according to the manufacturer's instructions. A total of 1 × 10^7^ HGC‐27 cells were harvested and lysed in RIP lysis buffer containing protease inhibitor cocktail and RNA inhibitor; the supernatants were collected after centrifugation for 10 min at 13000 rpm, and then incubated with Dynabeads protein G conjugated with anti‐IgG or anti‐AGO2 using a Magna RIP kit (Merck, USA). RIP‐Seq service was provided by Cloud‐Seq Biotech (Shanghai, China). Libraries were controlled for quality and quantified using the BioAnalyzer 2100 system (Agilent Technologies Inc.). Library sequencing was performed with 150 bp paired‐end reads.

### 2.12 Dual Luciferase Reporter Assay

HGC‐27 cells (Vector cells and YTHDF1 stable overexpression cells) were transfected with NF‐κB and Renilla expression plasmids (Yesem, China) per well using PolyJet transfection reagent in 24‐well plates. After 48 h of transfection, the cells were lysed with passive lysis buffer (Meilunbio, China), and reporter gene expression was assessed using a Dual Luciferase Reporter Assay System (Tecan, Switzerland).

### 2.13 Statistical Analysis

The data are reported as the mean ± the standard deviation (SD). In immunohistochemistry, qRT‐PCR, western blot analysis, colony formation assay, cell viability assay, Transwell migration experiment, migration assay, flow cytometry, and other experiments, the experimental groups were all two groups, and the student's *t*‐test was used to compare the differences between the two groups. The chi‐squared test was performed on the immunohistochemistry of the cancer and the count data (*χ* ^2^) of clinical parameters. The in vitro tests were conducted in triplicate, with a minimum of three separate trials. The clinical data was analyzed using the Kaplan–Meier survival curves. The variance between groups was statistically compared. If *p* < 0.05, all differences were considered statistically significant. * indicates *p* < 0.05, ** indicates *p* < 0.01, *** indicates *p* < 0.001, and **** indicates *p* < 0.0001. To address the issue of multiple testing, the *p* values were modified using the Benjamini–Hochberg false discovery rate adjustment. ImageJ was used for data analysis of experimental images for western blot analysis, colony formation assay, Transwell migration experiment, and migration experiment. SPSS Statistics 27 (IBM) and GraphPad Prism 8.0 were used for statistical analysis of all the above experimental data.

## 3 Results

### 3.1 Database Analysis Shows That Expression of YTHDF1 Is Markedly Greater in GC Than in Healthy Tissues and Affects Patient Prognosis

**FIGURE 1:**
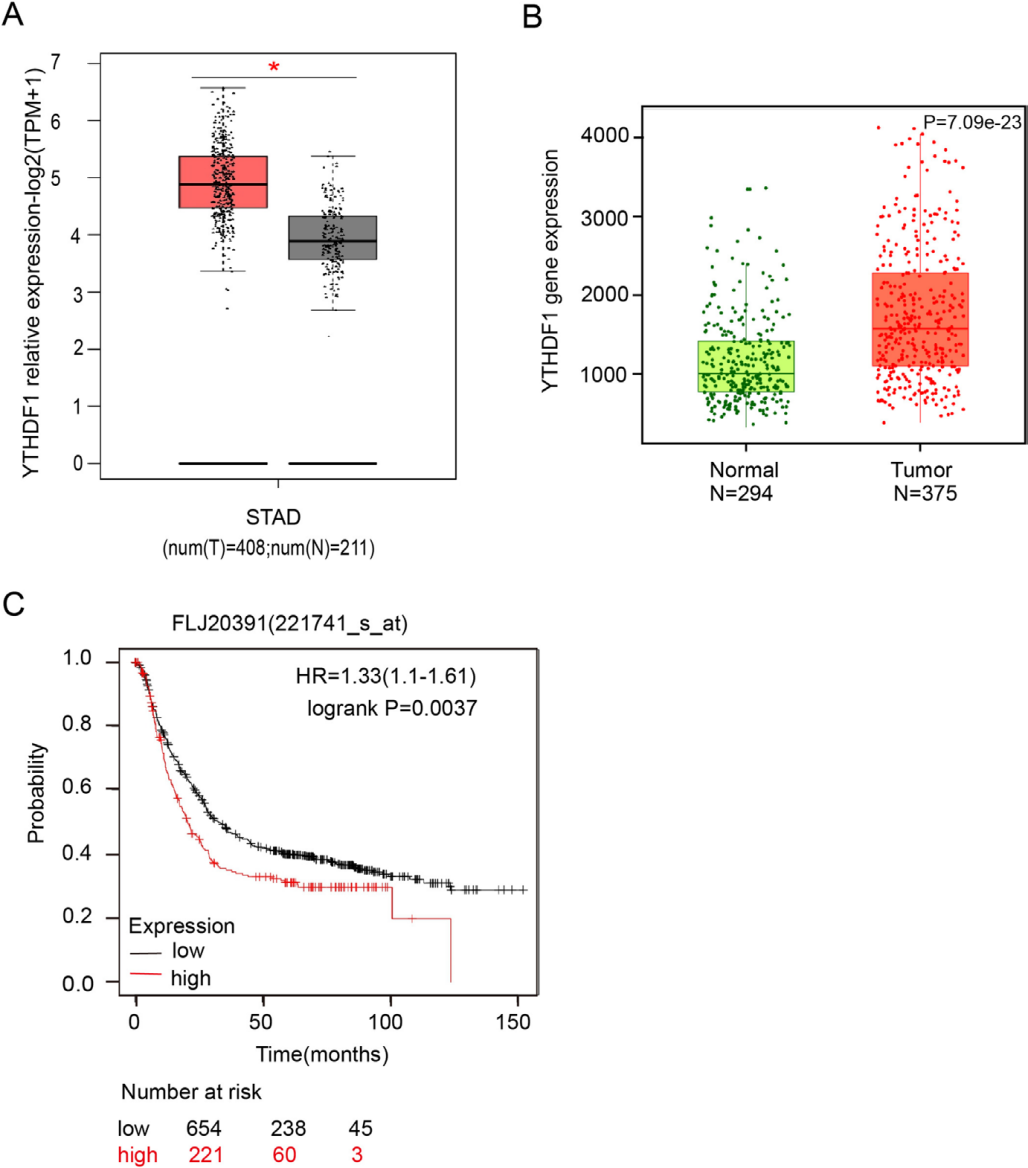
Gastric cancer (GC) is extremely associated with YTHDF1. (A) *YTHDF1* mRNA expression levels in GC tissues and normal tissues were analyzed using the GEPIA database, *p < 0.05. (B, C) The expression levels of YTHDF1 mRNA in GC tissues and normal tissues were analyzed using the Kaplan–Meier Plotter database, and the patients in the high‐expression category for YTHDF1 had a worse survival prognosis, ****p < 0.0001.

To explore the status of YTHDF1 in GC, we first applied the GEPIA database query and analysis. The comparison of GC tissue (*T* = 408) and paracancerous stomach tissues (*N* = 211) demonstrated that YTHDF1 was more highly expressed in GC tissue than in paracancerous stomach tissues (Figure 1A). Secondly, Kaplan–Meier plotter data analysis was implemented. The comparison of GC tissue (*T* = 375) and normal gastric tissue (*N* = 294) was conducted using RNA‐sequencing data, and the results indicated that YTHDF1 was substantially upregulated in GC tissue compared to normal gastric tissue (Figure 1B). Kaplan–Meier Plotter bioinformatics query and data analysis were used to investigate the correlation between YTHDF1 and patient survival prognosis. The findings indicated that patients in the high‐expression category for YTHDF1 (*n* = 221) had a worse survival prognosis than patients in the low‐expression category (*n* = 654) (Figure 1C).

### 3.2 The YTHDF1 Level Detected in Clinical Samples Is Substantially Higher in GC Compared to Natural Tissues and Affects Patient Prognosis

**FIGURE 2:**
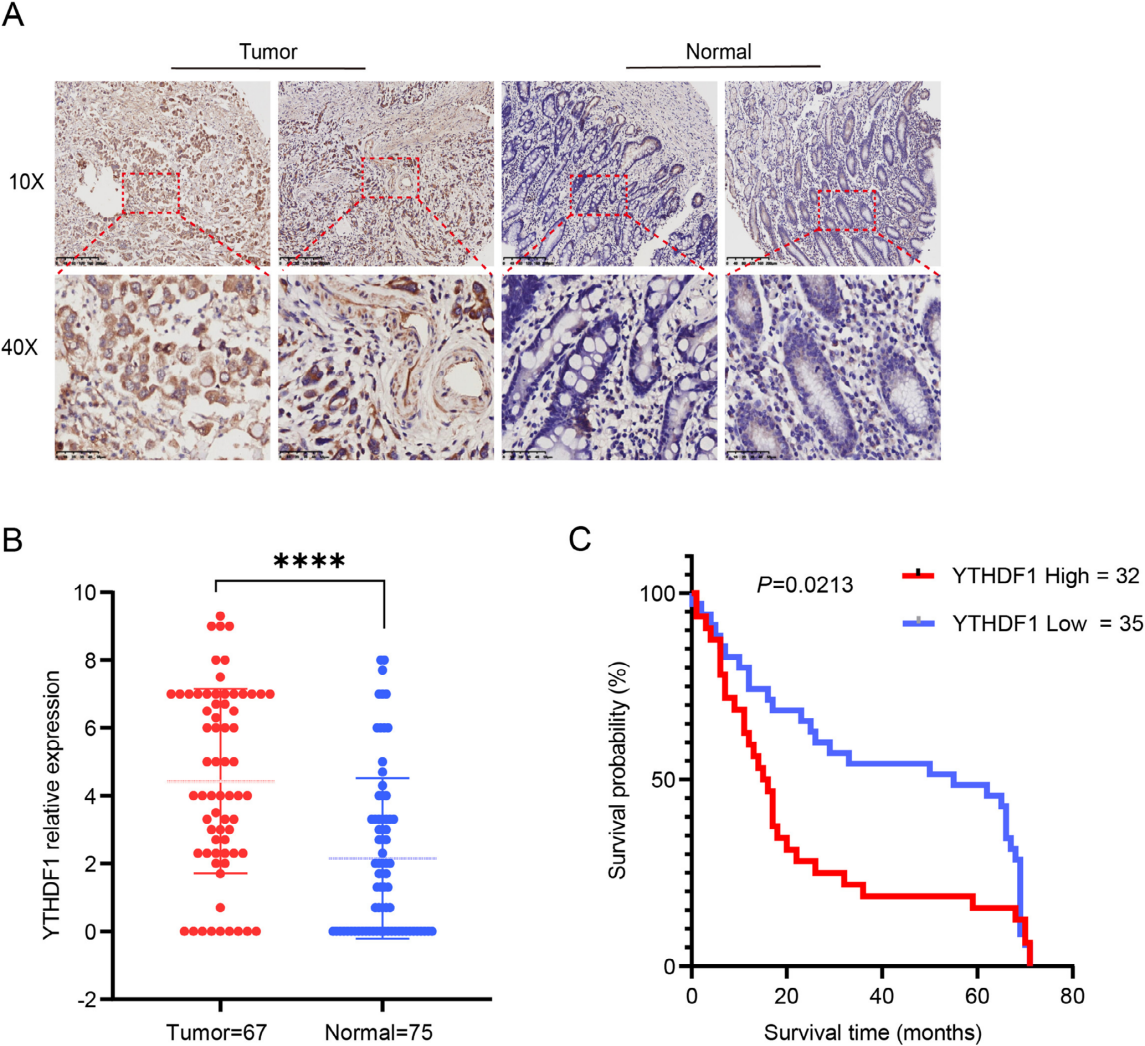
YTHDF1 is highly expressed in gastric cancer (GC) tissues. (A) YTHDF1 expressed in GC and paracancerous tissue, as detected by immunohistochemistry (IHC). (B) Pathology microarray IHC results suggest that YTHDF1 is highly expressed in GC tissue, ****p < 0.001. (C) Pathological microarray IHC results show that the prognosis of the high‐expression group is significantly different from that of the low‐expression group, *p < 0.01.

YTHDF1 was elevated in GC, indicating that YTHDF1 influences the incidence and progression of GC. In order to confirm this theory, we proceeded to use IHC to identify the presence of YTHDF1 in GC and adjacent non‐cancerous tissues on the GC pathological tissue chips. The IHC score was determined as follows: in each sample, we randomly selected three high‐power microscope fields, and the score was calculated by multiplying the intensity of color and the proportion of cells that are positive out of the total number of cells. The scores of the three high‐power microscope fields were calculated, and the final result was determined by calculating the average value. A score of 0 was represented as negative (−), scores 1–4 were considered moderately positive (+), scores 5–8 were classified as positive (++), and scores 9–12 were categorized as very positive (+++). The IHC score was used to categorize the GC and paracancerous tissues into two categories: the low‐expression category (score ≤ 4; *n* = 35) and the high‐expression category (score > 4; *n* = 32) (Figure 2A). The findings indicated that the expression level of YTHDF1 in GC tissues (*n* = 67) was markedly elevated compared to that in adjacent non‐cancerous tissues (*n* = 75) (Figure 2B). Patients with elevated YTHDF1 expression had a worse prognosis for survival, as demonstrated by Kaplan–Meier survival analysis (Figure 2C). In order to investigate the connection between YTHDF1 and the clinical parameters of patients, a correlational analysis (*χ* ^2^) was performed to assess the correlation between the expression of YTHDF1 and clinical parameters by SPSS 27 (IBM). The findings demonstrated a positive correlation between the expression level of YTHDF1 and lymph node metastasis, as well as the expression of PD‐1 and PD‐L1 in the tumor tissues of GC patients. However, no correlation was seen between the expression level of YTHDF1 and patient age, gender, tumor size, vascular invasion, nerve invasion, or tumor location (Table 1).

**TABLE 1:** Results of correlation between YTHDF1 and clinical parameters in 67 patients with GC.

Variables	Number of patients	YTHDF1		*χ* ^2^	*p*
		Low	High		
Gender				0.651	0.420
Male	39	22 (56.4%)	17 (43.6%)		
Female	28	13 (46.4%)	15 (53.6%)		
Age (years)				0.109	0.742
< 60	18	10 (55.6%)	8 (44.4%)		
≥ 60	49	25 (51.0%)	24 (49.0%)		
Tumor size				0.324	0.851
T2	9	4 (44.4%)	5 (55.6%)		
T3	40	17 (52.5%)	24 (47.5%)		
T4	16	7 (56.3%)	8 (43.8%)		
Node metastasis				12.954	0.005
N0	15	12 (80.0%)	3 (20.0%)		
N1	5	5 (100.0%)	0 (0.0%)		
N2	17	7 (41.2%)	10 (68.8%)		
N3	30	11 (36.7%)	19 (63.3%)		
TNM stage				5.847	0.119
I	4	3 (75.0%)	1 (25.0%)		
II	17	12 (70.6%)	5 (29.4%)		
III	44	19 (44.2%)	24 (55.8%)		
IV	1	0 (0.0%)	1 (100%)		
Nerve invasion				0.029	0.864
Positive	12	6 (50.0%)	6 (50.0%)		
Negative	55	29 (52.7%)	26 (47.3%)		
Tumor location				0.085	0.770
Gastric antrum	26	13 (50.0%)	13 (50.0%)		
Others	41	22 (53.7%)	19 (46.3%)		
Vascular invasion				1.675	0.196
Positive	24	10 (41.7%)	14 (58.3%)		
Negative	43	25 (58.1%)	18 (41.9%)		
PD‐1				7.091	0.008
< 1%	16	13 (81.3%)	3 (18.8%)		
≥ 1%	51	22 (41.3%)	29 (56.9%)		
PD‐L1				3.939	0.047
< 1%	13	10 (76.9%)	3 (23.1%)		
≥ 1%	54	25 (46.3%)	29 (53.7%)		

### 3.3 YTHDF1 Enhances the Proliferation and Migration of GC Cells While Suppressing the Programmed Cell Death of GC Cells

**FIGURE 3:**
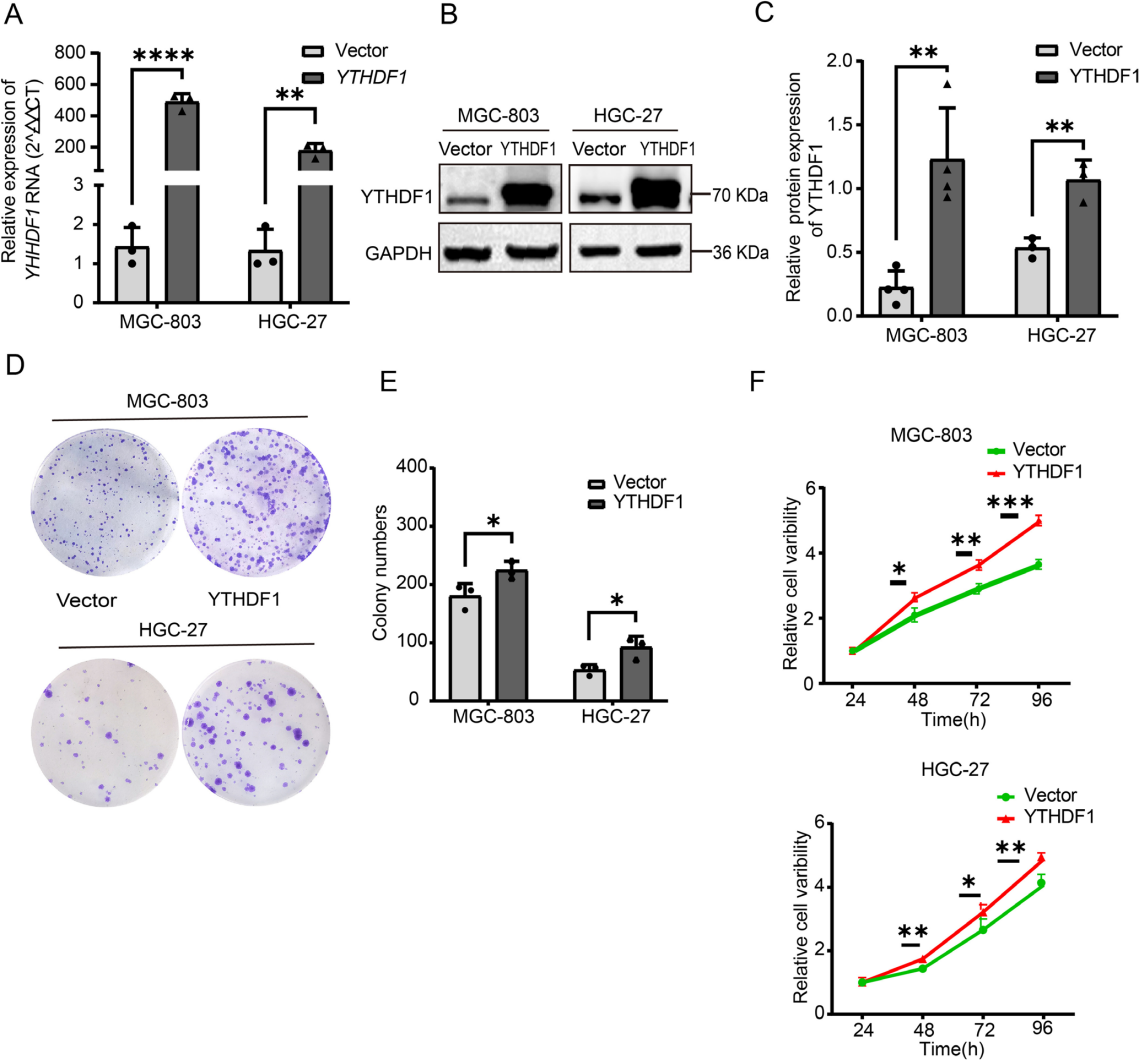
YTHDF1 overexpression promotes the proliferation and colony formation of gastric cancer cells and inhibits their apoptosis. (A) Expression of *YTHDF1* gene transcripts in MGC‐803 and HGC 27 cells was analyzed by Q‐PCR in overexpression and control groups. (B, C) Protein levels of YTHDF1 in GC cell lines determined by western blotting. The GAPDH gene was used as a reference gene. The bars show the mean ± SD of *YTHDF1* gene overexpression from three independent experiments. (D, E) Colony formation assays of GC cells with YTHDF1 overexpression and controls. (F) CCK8 assays of GC cells with YTHDF1 overexpression and controls.

We introduced YTHDF1 empty vector plasmid (Vector) and YTHDF1 overexpression plasmid (YTHDF1) into GC cells using stable transfection. The cells were cultivated in a six‐well plate until they reached a density of 60%–70%. For each well, 2 μg of the transfection plasmid was used. After 48 h of transfection, MGC‐803 GC cells were subjected to screening using a concentration of 1 μg/mL puromycin, while HGC‐827 GC cells were screened using a concentration of 0.5 μg/mL puromycin. The medium was replaced every other day, and puromycin was given to the stably transfected cells for 4 days. The cells that were amplified thereafter were confirmed using qRT‐PCR and western blot analysis (Figure 3A–C).

**FIGURE 4:**
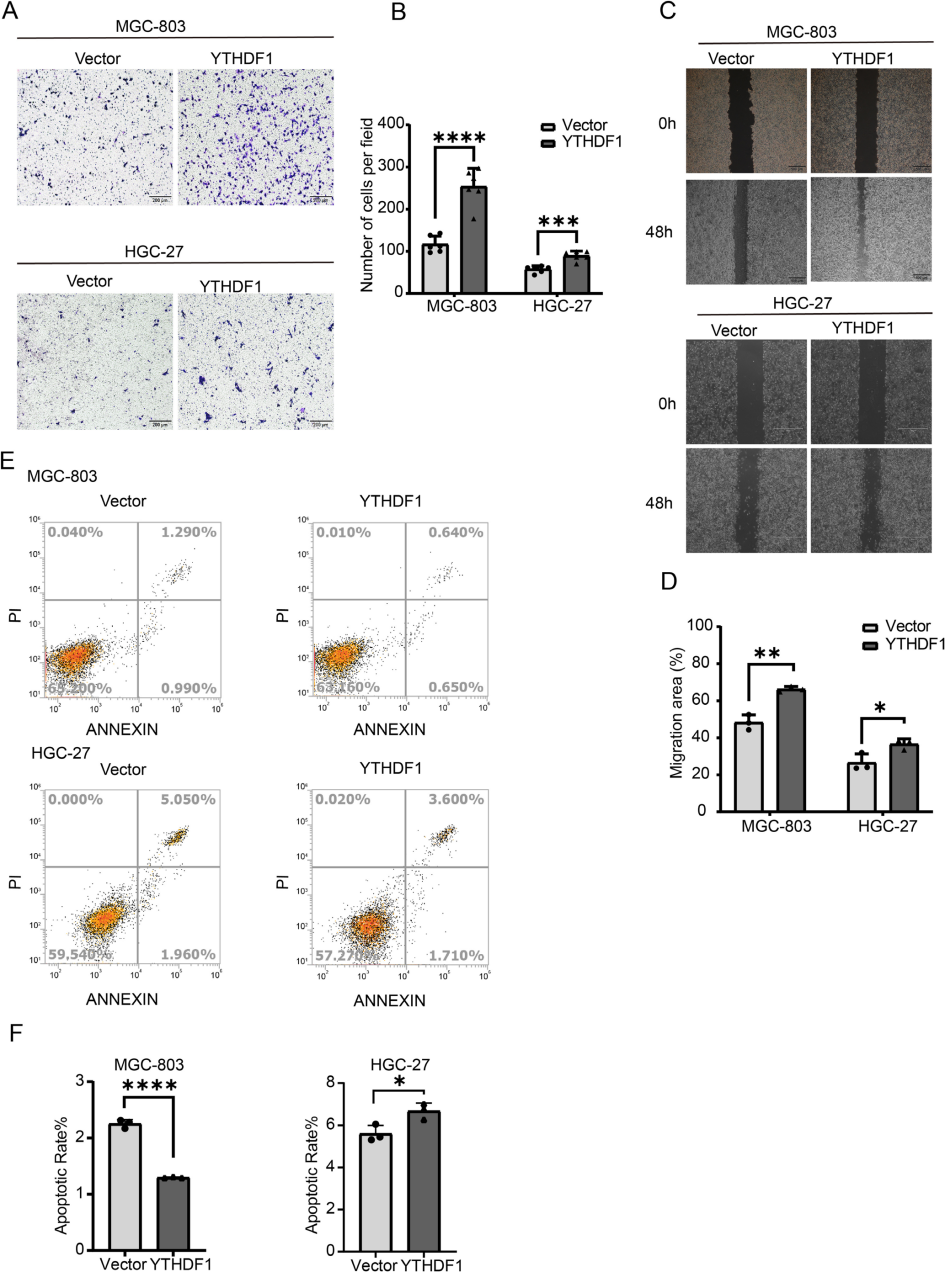
Overexpression of YTHDF1 promotes gastric cancer invasion and metastasis. (A, B) The migration of GC cells overexpressing YTHDF1 and controls was assessed. Scale bar represents 200 μm. In the bar graph on the right, the number of migrating cells is quantitatively compared for each field. (C, D) Wound healing analysis was performed to assess the effect of YTHDF1 on cancer cell migration capacity and showed relative mobility (mean ± SD, *n* = 3, unpaired *t* test). The bar graph on the right shows the quantification of the 0‐h and 48‐h wound healing assays. (E, F) Cell apoptosis is inhibited by overexpression of the YTHDF1 gene in flow cytometry assays. **p < 0.01, ***p < 0.001 versus NC‐OE group, *n* = 3 (Student's *t* test).

The stably transformed GC cell line was constructed to study whether YTHDF1 affects GC proliferation This study used a colony formation assay to assess the proliferation capacity of the stably transformed strains, namely, the Vector group and the YTHDF1 group. Each well of a six‐well plate was cultivated with 1000 cells, which were then fixed and stained for approximately 2 weeks (Figure 3D,E). In the cell viability assay, GC cells in the Vector group and the YTHDF1 group were treated with trypsin and placed in a 96‐well plate with 4000 cells per well. The optical density (OD) of the cells was measured at 24, 48, and 72 h. The results showed that the proliferation ability of GC cells in the YTHDF1 group was significantly higher than that in the vector group (Figure 3F). GC cells in the Vector group and the YTHDF1 group were digested with trypsin in the Transwell experiment. A total of 50,000 cells were cultured in each migration chamber and fixed and stained after 24 h. In the migration assay, GC cells in the Vector group and the YTHDF1 group were cultured in serum‐free medium after scratching when the cell density reached 100%, and photographs were taken at 0 and 48 h. The photographs were taken at 10× magnification. The findings indicated that the migratory capacity of the YTHDF1 group was considerably superior to that of the Vector group (Figure 4A–D). Cell flow cytometry was utilized to identify apoptosis in both the Vector group and the YTHDF1 stably transformed strain group. The findings indicate that YTHDF1 has the ability to suppress apoptosis in GC cells (Figure 4E,F).

### 3.4 YTHDF1 Can Affect the Expression of RELA Protein, but It Does Not Affect the Transcription of *RELA* RNA. YTHDF1 Works by Affecting the Translation of RELA

**FIGURE 5:**
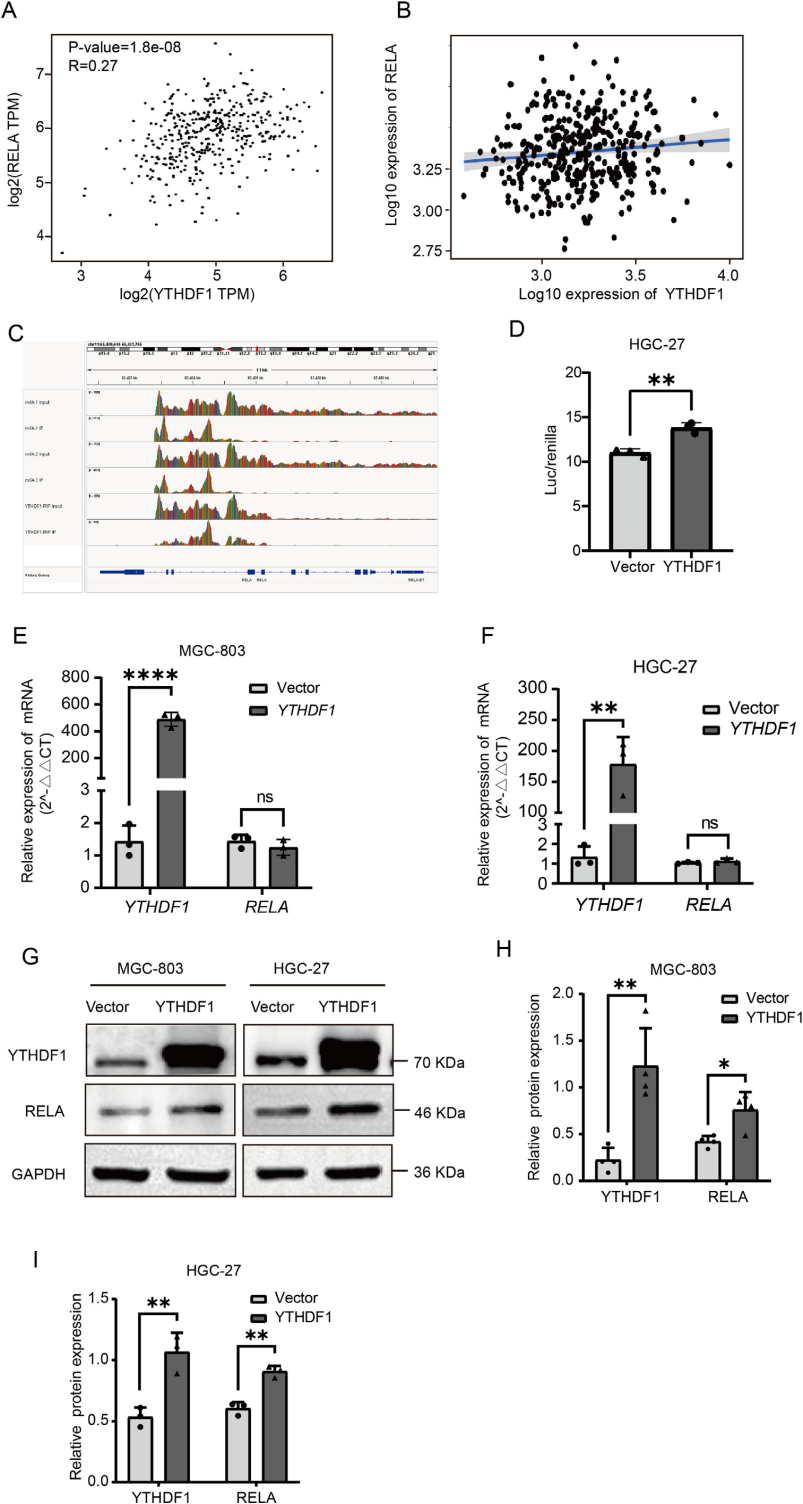
In gastric cancer (GC) cells, the m6A mutation of the crucial transcription factor RelA in the NF‐κB pathway is identifiable by YTHDF1. (A) GEPIA database query and analysis show that the expression of YTHDF1 positively correlates with RELA expression in GC. ****p < 0.0001. (B) Positive correlation between YTHDF1 expression and RELA expression in TCGA data. (C) Conclusions of the RIP‐seq experiment using m6A‐RIP and YTHDF1 antibodies demonstrate that the IGV software reveals the association of m6A and YTHDF1 with the *RELA* gene. (D) Following the transfection of HGC‐27 cells (both control and YTHDF1 stable overexpression variants) with NF‐κB and Renilla expression plasmids for 48 h, the fluorescence signal in the YTHDF1 stable overexpression cells was augmented. **p < 0.01. (E, F) GC cells stably transfected with empty vector plasmids (NCs) and overexpressed plasmids (OEs) were analyzed by RT‐qPCR. (G–I) Western blotting showing that overexpression of YTHDF1 can increase the expression level of RELA. **p < 0.01, ****p < 0.0001 versus NC‐OE group, *n* = 3 (Student's *t* test).

We next explored the mechanism and pathway of YTHDF1 in GC. The query and analysis of the GEPIA database and Kaplan–Meier Plotter database showed that YTHDF1 and RELA positively correlated in GC (Figure 5A,B). In order to confirm the connection between YTHDF1 and RELA, we performed m6A‐RIP experiments utilizing m6A antibodies and RIP‐seq experiments employing YTHDF1 antibodies. The gene enrichment analysis of the sequencing results indicated that *RelA* mRNA is located in the intersection of the two sets of enriched genes (Figure 5C). Employing dual‐luciferase assays to confirm that YTHDF1 can activate the NF‐κB pathway (Figure 5D). We used qRT‐PCR to measure the expression of *RELA mRNA* at various *YTHDF1* levels. The results showed that there was no significant change in *RELA mRNA* in GC cells at different levels of *YTHDF1* (Figure 5E,F). The technique of western blot analysis was used to identify the presence of RELA protein at various levels of YTHDF1. At different YTHDF1 levels, the findings revealed notable variations in RELA protein levels inside GC cells (Figure 5G–I).

### 3.5 YTHDF1 Promotes GC Proliferation by Regulating Protein Translation of RELA

**FIGURE 6:**
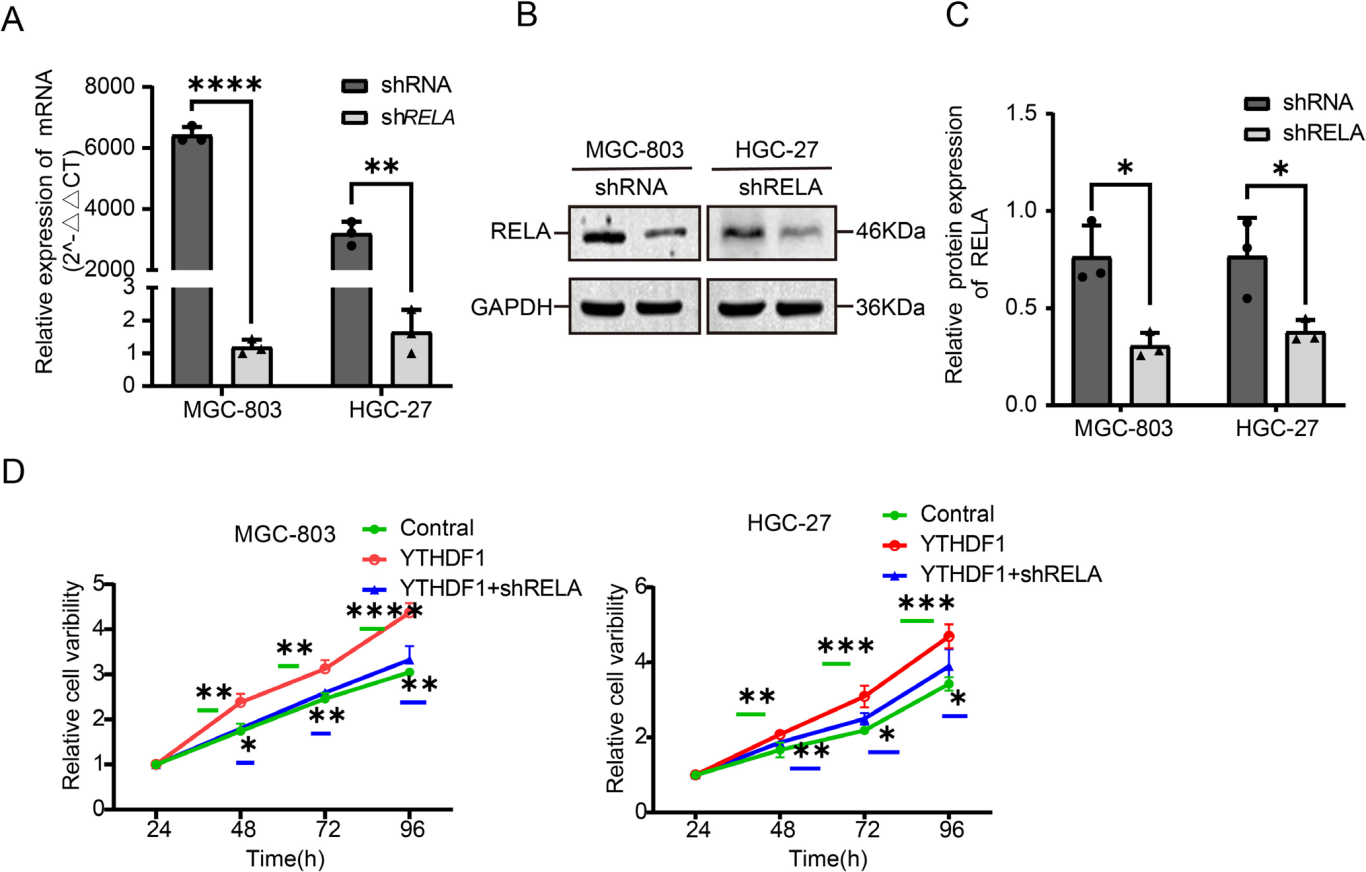
Effect of the knocked‐down RELA gene on the overexpressed YTHDF1 gene in gastric cancer cells. (A) Expression of knockdown and control *RELA* genes in MGC‐803 and HGC 27 cells, as analyzed by Q‐PCR. (B, C) The protein levels of the RELA gene knockdown group and the control group in GC cell lines were determined by Western blotting. GAPDH was used as an internal reference gene. (D) CCK8 assay of GC cells with YTHGF1 overexpression, YTHDF1 with RELA knockdown, and their controls.

In further experiments, *RELA* empty vector plasmid (sh*RNA*) and knockdown plasmid (sh*RELA*) were transfected. The qRT‐PCR method was used to confirm the expression levels of *REAL mRNA* (Figure 6A), while western blot analysis was performed to confirm the expression levels of RELA protein (Figure 6B,C). In subsequent studies, the stably transfected cell lines of the Vector group and YTHDF1 group were superimposed and transfected with RELA empty vector (sh) group and knockdown (shRELA) group to obtain vector+shRNA (Control), YTHDF1, and YTHDF1 + shRELA for functional experimental studies. For the cell viability assay, a total of 5000 cells were cultivated in each well of a 96‐well plate. The optical absorbance (OA) value of the cells was determined at 24, 48, and 72 h. The findings demonstrated that suppressing RELA effectively counteracted the proliferative effects induced by overexpression of YTHDF1 (Figure 6D).

### 3.6 YTHDF1 Promotes GC Migration and Inhibits Apoptosis by Regulating RELA Protein Production

**FIGURE 7:**
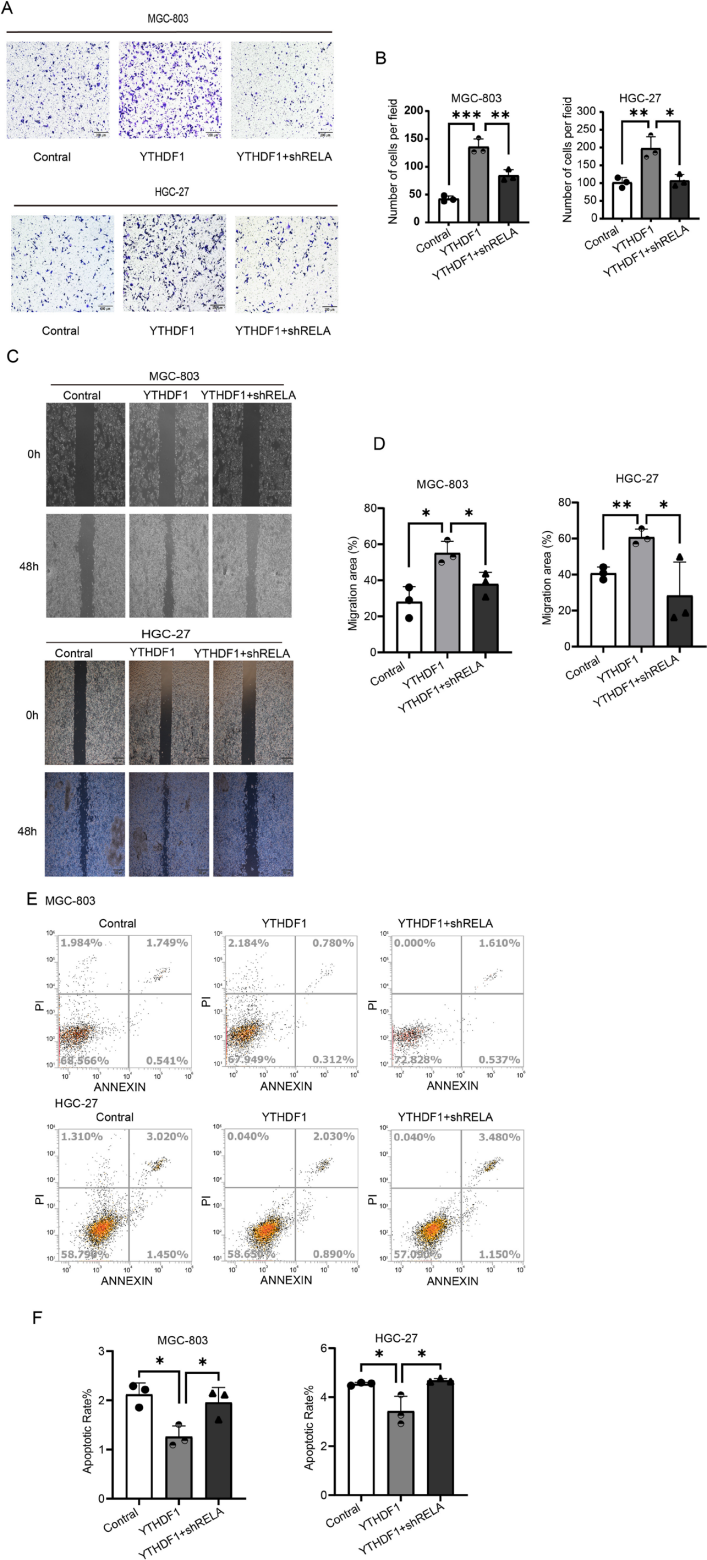
RELA knockdown antagonizes the effects of overexpressed YTHDF1 in gastric cancer (GC). (A, B) An assay for migration of GC cells overexpressing YTHDF1, overexpressing YTHDF1 with RELA knockdown, and controls. Scale bar represents 200 μm. The bar chart on the right shows a quantitative comparison of the number of migration units for each field. (C, D) Wound healing assays were performed to assess the migration capacity of gastric cancer cells transfected with YTHDF1 overexpression plasmid, YTHDF1 overexpression with RELA knockdown plasmid, and control plasmids; relative mobility is shown (mean ± SD, *n* = 3, unpaired *t* test). The bar graph on the right shows the quantification of the 0‐h and 48‐h wound healing assays. (E, F) Flow cytometry assays show that knockdown of RELA gene could reverse the inhibitory effect of overexpressed YTHDF1 gene on apoptosis of gastric cancer cells.

In the following study, 50,000 cells were cultured in each migration chamber in the transwell migration experiment and fixed and stained after 24 h (Figure 7A,B). During the migration assay, cells with a density over 90% were grown in a medium without serum following the scratching process. Photographs were captured at both 0 and 48 h (Figure 7C,D). The findings demonstrated that suppressing RELA effectively counteracted the proliferative effects induced by YTHDF1 overexpression. Apoptosis detection was performed using cell flow cytometry in both the Vector group and the YTHDF1 stably transfected strain group. The findings demonstrated that suppressing RELA effectively counteracted the apoptotic‐promoting function of YTHDF1 in GC cells (Figure 7E,F).

## 4 Discussion

The research we conducted revealed that YTHDF1 exhibits significant upregulation in GC, indicating that YTHDF1 functions as an oncogenic gene in GC. Moreover, patients with a higher expression of YTHDF1 have a worse prognosis for survival, which also indicates that YTHDF1 may be an indicator for judging the prognosis and relapse of GC.

Targeted treatment is a crucial method for treating cancer and has also been acknowledged in GC research [24]. In this study, we performed a correlation analysis of clinical sample parameters and found that in individuals with clinical GC, there was a significant correlation between the expression level of YTHDF1 and lymph node metastases. While researching breast cancer [25], research has demonstrated that YTHDF1 is a causative factor in cancer, namely in promoting the growth, invasion, and movement of breast cancer cells [26]. Combined with the positive correlation between YTHDF1 and lymph node metastasis, this suggests that YTHDF1 may promote the metastasis of GC. This biological function was also confirmed through cell experiments.

Based on previous studies, YTHDF1 has been shown to facilitate the proliferation of lung cancer, endometrial cancer, and other types of cancers [27, 28]. The research revealed that METTL3 actively facilitates the growth and development of colonies in non‐small cell lung cancer (NSCLC) cells, namely via its dependence on m6A‐YTHDF1 [27]. Additionally, several researchers have determined that in endometrial cancer, the YTHDF1 enhances the growth of tumors by controlling the ERK/NF‐κB/AKT signaling cascade via the PAPPA/IGFBP4 axis [28]. This experimental investigation also revealed that YTHDF1 can enhance GC cell proliferation. Prior research has shown that suppressing YTHDF1 leads to apoptosis in cervical carcinoma [25]. Similarly, our results suggest that excessive expression of YTHDF1 in GC cells inhibits apoptosis. Numerous studies support the notion that YTHDF1 promotes tumor growth while suppressing apoptosis. Therefore, based on this study's results, the involvement of YTHDF1 in boosting GC cell proliferation and preventing apoptosis is well apparent.

Earlier scholars have confirmed that YTHDF1 can promote the proliferation and migration of GC [29], which is consistent with the conclusions of this study. The innovation of this study is complementary to the study of YTHDF1 for GC apoptosis, which found that it not only promotes tumor growth but also inhibits cell apoptosis. It was discovered that the path of adjustment was different from that of prehistoric scholars. During the pathway exploration research, we found a positive correlation between YTHDF1 and RELA. YTHDF1 can regulate the translation of RELA protein without affecting its transcription. The transcription factor RELA is a key player in the NF‐κB pathway, where it has a significant impact by increasing the expression of molecules that promote inflammation and cancer. This process contributes to tumor development and treatment resistance, ultimately activating various mechanisms such as cell proliferation, apoptosis, tumor metastasis, the pro‐inflammatory response, and metabolic reprogramming to drive tumor progression [22, 23]. YTHDF1 is a versatile reader for m6A modifications, and its main function is to mediate protein translation of downstream genes. According to a recent study on colon cancer, YTHDF1 can promote the translation of RELA, participate in the NF‐κB pathway, and promote tumor development [30]. In this study, we also confirmed that YTHDF1 can function by regulating the translation of RELA, and the mechanism involves the NF‐κB pathway.

A large body of literature suggests that YTHDF1 promotes various tumors. The results of this study were based on bioinformatics analysis, pathological tissue sections, and in vitro experiments. It can be inferred that YTHDF1 promotes cell division and movement in GC. The advantages of this study are that YTHDF1 has been supplemented with its impact on GC apoptosis and its involvement in the new regulation mechanism pathway of the NF‐κB pathway has been explored by facilitating the translation of RELA.

However, there are still shortcomings in the completeness of the experiment. This experiment was based on bioinformatics analysis, pathological tissue sections, and in vitro experiments. However, the phenotype could not be verified at the level of animal (mouse) experiments. There are still certain limitations. Therefore, additional animal experiments are needed to further confirm the conclusions.

## 5 Conclusion

The level of YTHDF1 expression in GC is much increased compared to its expression in nearby tissues. Higher expression of YTHDF1 in cancer is associated with poorer survival. YTHDF1 can be used as an indicator for diagnosing GC and judging prognosis. YTHDF1 expression level positively correlates with lymph node metastasis and expression of PD‐1 and PD‐L1. YTHDF1 can enhance the development and proliferation of GC cells while preventing apoptosis. Furthermore, it facilitates GC growth by controlling RELA translation and stimulating the NF‐κB pathway.

## Author Contributions

**Yangyuan Huang:** investigation (lead), methodology (lead), writing – original draft (lead). **Shihao Liang:** validation (equal), writing – review and editing (equal). **Liping Li:** software (equal), validation (equal). **Qingyu Zeng:** validation (equal). **Bin Li:** conceptualization (lead), data curation (lead), formal analysis (lead), funding acquisition (lead), investigation (equal), methodology (supporting), project administration (lead).

## Ethics Statement

This study was performed in line with the principles of the Declaration of Helsinki. The exempt was granted by the Ethics Committee for the use of the HStmA180Su19 tissue microarray. It should be noted that all of the above samples have received Shanghai Xinchao Biotechnology Co. Ltd. Ethics Committee approval and informed consent.

## Conflicts of Interest

The authors declare no conflicts of interest.

## Supporting information


Data S1.

## Data Availability

Data will be made available on request.
